# Supplementary material for: Cryo-focused ion beam preparation of perovskite based solar cells for atom probe tomography
Source: PLoS One. 2020 Jan 16;15(1):e0227920. doi: 10.1371/journal.pone.0227920 (PMC6964857; doi:10.1371/journal.pone.0227920)
Supplement: Attachment — Submitted filename: Recommendation.docx [file pone.0227920.s001.docx]

Dear editor:

The manuscript entitled” Cryo-Focused Ion Beam Preparation of Perovskite based solar cells for Atom Probe Tomography” works on using Cryo-FIB to prepare samples for hybrid perovskite solar cells. The organization of the data and language in this manuscript is well organized and clear expressed. The detailed operation process and the results obtained from the measurement are helpful for the understanding of perovskite materials. Thereby, I do recommend publishing this manuscript after minor revision.

1. Are there some reports on sample preparation of hybrid perovskite film? If yes, please make comparison to confirm the advantages with cryo.
2. The fullerene layer is observed as discreet islands as mentioned in the manuscript. Does the so-called island formed during the sample preparation using FIB? Please supply the cross-sectional images for samples prepared by simple break rather than FIB.
